# LAMT: Lightweight and Anonymous Authentication Scheme for Medical Internet of Things Services

**DOI:** 10.3390/s25030821

**Published:** 2025-01-30

**Authors:** Hyang Jin Lee, Sangjin Kook, Keunok Kim, Jihyeon Ryu, Youngsook Lee, Dongho Won

**Affiliations:** 1 Department of Electrical and Computer Engineering, Sungkyunkwan University, Suwon-si 16419, Republic of Korea; hyangjin.lee@gmail.com (H.J.L.); sangjinkook@gmail.com (S.K.); kimkeunok@gmail.com (K.K.); dhwon@security.re.kr (D.W.); 2 School of Computer and Information Engineering, Kwangwoon University, Seoul-si 01897, Republic of Korea; 3 Department of Computer Information Security, Howon University, 64 Impi-myeon, Howondae 3-gil, Gunsan-si 54058, Republic of Korea; ysooklee@howon.ac.kr

**Keywords:** medical IoT system, lightweight authentication, anonymous user authentication, sensor authentication

## Abstract

Medical Internet of Things (IoT) systems can be used to monitor and treat patient health conditions. Security and privacy issues in medical IoT services are more important than those in any other IoT-enabled service. Therefore, various mutual authentication and key-distribution schemes have been proposed for secure communication in medical IoT services. We analyzed Hu et al.’s scheme and found that an attacker can impersonate legitimate sensor nodes and generate illegitimate session keys using the information stored in the sensor node and the information transmitted over the public channel. To overcome these vulnerabilities, we propose a scheme that utilizes physically unclonable functions to ensure a secure session key distribution and increase the computational efficiency of resource-limited sensor nodes. In addition, the proposed scheme enhances privacy protection using pseudonyms, which we prove using a formal security analysis tool, ProVerif 2.05.

## 1. Introduction

Network services based on sensors are increasing owing to the expansion of utilization fields as a result of the development of sensor technology and the increase in network services due to COVID-19 [1]. Recently, medical devices have been connected to network services worldwide, and medical Internet of Things (IoT) services based on sensors, such as remote medical treatment and the monitoring of patients’ health status by doctors, have been increasing [2]. However, in the medical field, security issues are among the most significant factors hindering the expansion of network services that use medical devices. In particular, information transmitted through medical devices not only requires sensitive privacy but can also cause serious problems for the patient’s health if tampered with, which is why medical IoT environments require strong security protocols [3]. Therefore, government departments in charge of medical device licensing have set high-level requirements for cybersecurity and privacy for medical devices distributed in their countries.

IoT environments used in the healthcare sector often collect and transmit large amounts of individual medical data [3]. The collection of such sensitive data in large volumes poses risks of unauthorized access, use, or disclosure [4]. To ensure secure data management, many devices used in the healthcare field employ methods such as encryption, access control, identity management, and user authentication [5].

Among the various security requirements of IoT service environments based on medical devices, user authentication and privacy protection are the most important. Various studies have been proposed to address user authentication and privacy protection for IoT devices [6,7,8]. In 2021, a user authentication system integrated with blockchain was proposed, which automatically manages the creation, deletion, and addition of multiple data [6]. Additionally, a user authentication system utilizing hospital cloud servers was introduced [7]. In 2022, Hu et al. [8] presented a user authentication scheme between users and sensor nodes that employs two-factor security using the user’s password and smart card. Their scheme features the use of elliptic curve operations to protect values.

In this study, we analyzed the vulnerabilities of the scheme of Hu et al. [8] based on a general system model and attack model in medical IoT services. We found that an attacker can impersonate legitimate sensor nodes and generate illegitimate session keys using the information stored in the sensor node and the information transmitted over the public channel. To overcome these vulnerabilities, we propose a lightweight and anonymous user authentication scheme.

The contributions of the proposed scheme are summarized as follows:

- We thoroughly analyzed the scheme proposed by Hu et al. and identified the following three issues. First, attackers can extract critical authentication values from the data transmitted by sensor nodes and over public channels in Hu et al.’s scheme. Second, these extracted values can be used to perform impersonation attacks on the sensor node. Lastly, attackers can share sessions with the user. To address these three issues, we propose a new scheme, LAMT, in this study.
- The proposed scheme does not store the information used to generate the session key on the user’s mobile device or sensor node. Thus, even if an attacker physically obtains the mobile device or sensor node, it is impossible to perform an impersonation attack.
- The proposed scheme minimizes the computation at sensor nodes by using a physically unclonable function (PUF). In the proposed scheme, a challenge–response pair of the PUF is transmitted over a private channel, and only challenges are transmitted over a public channel; thus, it is resistant to PUF modeling attacks.
- The proposed scheme ensures anonymity and untraceability by using pseudonyms that change from session to session instead of user and sensor node identities. It also ensures forward secrecy, mutual authentication, and is resistant to man-in-the-middle and PUF modeling attacks. Its security was confirmed using ProVerif, a formal security analysis tool. The proposed scheme achieved an average computational cost reduction of 985.73% when compared with the latest research [8,9,10,11] in terms of performance.

The remainder of this paper is organized as follows. In Section 3, we present the hash functions of the system and attack models. The target scheme is introduced in Section 4. In Section 5, we describe the limitations of the proposed scheme. Our scheme is described in Section 6. In Section 7, we present formal and informal security analyses. In Section 8, we present the performance analysis of the proposed scheme, and in Section 9, we discuss the results. Finally, we conclude this paper in Section 10.

## 2. Related Work

Recently, extensive research has been conducted on authentication schemes for wireless sensor networks (WSNs) used in medicine and healthcare. To provide high-strength security, theories have suggested the use of elliptic curve cryptography (ECC) or fuzzy extractors to extract biometric information.

In 2019, Ostad-Sharif et al. [12] proposed a user authentication scheme suitable for WSNs in IoT environments. In 2020, Chen et al. [13] identified design flaws in Ostad-Sharif et al. [12]’s scheme, highlighting issues with password-change functionality and time synchronization. Chen et al. [13] proposed an improved authentication scheme with enhanced security. Sahoo et al. [10] also identified design flaws in Ostad-Sharif et al. [12]’s scheme and proposed an ECC-based three-factor authentication scheme with enhanced security. However, in 2022, Hu et al. [8] revealed that Chen et al. [13]’s authentication method was vulnerable to offline password-guessing and impersonation attacks and failed to achieve perfect forward secrecy. Hu et al. [8] proposed a secure authentication scheme for WSNs in IoT environments using ECC. However, we found that Hu et al. [8]’s scheme is vulnerable to sensor impersonation attacks.

In 2018, Zhang et al. [14] proposed an authentication scheme for users and servers in health systems. However, in 2019, Aghili et al. [15] pointed out that Zhang et al. [14]’s scheme allowed user tracking and was vulnerable to desynchronization and DoS attacks. They proposed LACO [15], an approach in which a server is positioned between users and sensors to store data securely and allow users to share the data with the sensors. However, Amintoosi et al. [16] found that LACO [15] did not provide perfect forward secrecy and was vulnerable to sensor impersonation attacks; thus, they proposed a secure scheme called Slight [16]. In 2023, Wu et al. [9] demonstrated that Slight [16] was inaccurately designed with respect to session key values and susceptible to privileged-insider attacks, leading them to propose a new IoHT with enhanced security.

In 2019, Gao et al. [17] proposed a biometric-based scheme for WSNs. However, in 2021, Xue et al. [18] found that Gao et al. [17]’s scheme was vulnerable to offline password-guessing attacks and did not provide forward secrecy. Xue et al. [18] proposed an improved three-factor authentication scheme with enhanced security. In 2024, Huang [11] found that Xue et al. [18]’s scheme failed to provide anonymity and had a critical vulnerability in which a user’s private key could be extracted. To address these issues, Huang [11] proposed an enhanced user authentication scheme for WSNs that utilizes user biometric information and ECC for improved security.

## 3. Preliminaries

In this section, we first introduce the system model and the attack model of our proposed scheme, followed by the definitions and properties of the PUF used in our proposed scheme.

### 3.1. System Model

Our scheme has three entities: user, medical gateway (gateway), and medical IoT sensor node (sensor node).

1. Gateway: The gateway is a trusted entity that can know the identities of all users and sensor nodes, and all users and sensor nodes authenticate and communicate through the GWN. The GWN has powerful resources to perform complex computations.
2. User: The user is a legitimate entity who has access to a patient’s medical information. The user can access patient information using mobile devices (e.g., a smartphone or tablet) with limited resources.
3. Sensor node: In our scheme, the sensor node is an entity that collects the patient’s medical/health information and transmits it to the legitimate user through the GWN. The sensor node is typically a low-power device placed in or on the patient’s body.

IoT devices used in the medical field have limited computing power due to their nature, which significantly limits their ability to implement the advanced security protocols or complex communication protocols that were widely used in the past. This is a major cause of difficulties in building advanced systems for the safe transmission and processing of medical data [19].

The reasons for this can be found in the limited computational speed, memory capacity, and power usage requirements of IoT devices, which make it difficult to efficiently process or store large amounts of medical data collected in real time.

In addition, due to limitations in hardware resources, it is difficult to support various communication protocols simultaneously or process multiple tasks in parallel, and from a security perspective, lightweight security protocols or solutions with questionable safety must be used, which poses a risk of exposure to potential security vulnerabilities.

The minimum requirements for IoT devices in the proposed system are as follows:

1. Basic operational hardware: support for shift registers and arithmetic logic units for hash operations and execution of XOR operation units within XOR gate arrays or arithmetic logic units (ALUs) defined by application protocols.
2. Memory and storage: memory to store input data and intermediate results, ROM/flash memory to store protocol execution order and key information, and memory in the secure area to store important information.
3. A secure entropy source for security: a method for generating secure random numbers used by application protocols.
4. Interface for communication: providing a method for transmitting and receiving data required to implement application protocols.

### 3.2. Attack Model

The model assumes that the cryptographic primitives used in the protocol are secure and that the attacker has the following capabilities:

1. The attacker can intercept, delete, modify, store, and reply to any message exchanged over the public communication channel.
2. The attacker can physically capture the user’s mobile device and obtain some of the security parameters stored on the device.
3. The attacker can calculate the identity and password in polynomial time, but cannot gain the user’s identity, password, and information stored on the device at the same time.
4. The attacker can launch multiple attempts at attacks.

### 3.3. Physical Unclonable Function (PUF)

We used PUF technology to perform the authentication and session key distribution steps between the sensor nodes and gateways. A PUF can generate a unique response Rn for a specific challenge Cn by utilizing the characteristics of an IC chip and is used to implement methods for authenticating devices in physically insecure environments because of its advantage of being unclear. PUFs include optical PUFs, arbiter PUFs, and memory-based intrinsic PUFs. Optical PUFs are a representative example of optical PUFs. Optical PUFs create one-way functions through physical means, and challenge–response pairs (CRPs) are used only once to prevent replay attacks. However, optical PUFs require bulky external apparatus, resulting in high costs. CMOS-compatible APUFs (arbiter PUFs) exploit random interconnects and transistor gate time delays introduced during manufacturing. However, APUFs are based on a linear additive structure, making them vulnerable to various modeling attacks. Memory-based intrinsic PUFs refer to PUFs embedded in electronic devices during the manufacturing process. These are practical due to the ubiquity of memory in commercial electronic products. The PUFs we use are assumed to satisfy the following properties:

1. A PUF is a physically unclonable function. PUFs are fundamentally assumed to be resistant to cloning and modification. They are also assumed to be highly resistant to environmental factors and aging. In other words, an identical device cannot be made by cloning a PUF, and attempts to change a device containing a PUF will change the operation of the PUF and destroy it.
2. A PUF has the property of a one-way function. In other words, the challenge Cn cannot be found using the output response Rn of a specific PUF.
3. Different PUF1 and PUF2 output different responses Rn1 and Rn2 for the same challenge Cn.
4. For the same challenge Cn, one PUF outputs the same response Rn every time.
5. When multiple PUFs are used, the probability of collision is assumed to be low, meaning devices with identical responses are highly unlikely. Therefore, PUFs can be used in IoT devices to distinguish between a large number of devices effectively.

## 4. Review of Hu et al.’s Scheme

In this section, we introduce Hu et al. [8]’s scheme. The scheme proposed by Hu et al. [8] consists of three participants: the user, gateway node, and sensor node. The user and sensor node perform mutual authentication to share a session key. This scheme is divided into initialization, registration, and login and authentication phases. The details are as follows.

### 4.1. Initialization Phase

The gateway node GWN generates two secret keys $K_{GS}$ and $K_{GU}$ and creates the generator *P* of an elliptic curve. Subsequently, it computes the public key $pk=K_{GU}P$.

### 4.2. Registration Phase

This phase comprises user registration and sensor node registration. The details are as follows:

#### 4.2.1. User Registration Phase

To register with GWN, user $U_{i}$ follows these steps:

1. $U_{i}$ inputs their $ID_{i}$ and $PW_{i}$ and then generates a random number $r_{i}$. $U_{i}$ subsequently computes $A_{i}=h(ID_{i}\|PW_{i}\left\|r_{i}\right)$ and sends the message $\left\{ID_{i},\;A_{i}\right\}$ to GWN through a secure channel.
2. After receiving the message from $U_{i}$, GWN sets the expiration time $TE_{i}$ for the protocol. Then, using the public key of the sensor, it computes the temporary credential $TC_{i}=h(ID_{i}\|ID_{GWN}\|pk\|TE_{i})$ and $PTC_{i}=TC_{i}⊕A_{i}$. GWN stores $\left\{ID_{GWN},\;TE_{i},\;pk,\;h\left(\right),\;PTC_{i}\right\}$ with hash function h() in the smart card SC that will be sent to the user and transmits it to $U_{i}$ through a secure channel.
3. $U_{i}$ computes $TC_{i}=PTC_{i}⊕A_{i}$ and $B_{i}=TC_{i}⊕h\left(ID_{i}\|PW_{i}\right)$ and stores $B_{i}$ instead of storing $PTC_{i}$ in the smart card SC. Finally, SC contains $\left\{ID_{GWN},\;TE_{i},\;pk,\;h\left(\right),\;B_{i}\right\}$.

#### 4.2.2. Sensor Registration Phase

GWN selects the identity $SID_{j}$ of sensor node $S_{j}$. It then computes the temporary credential $TC_{j}=h\left(K_{GS}\|SID_{j}\right)$ and sends $\left\{SID_{j},\;TC_{j}\right\}$ through a secure channel. $S_{j}$ securely stores $\left\{SID_{j},\;TC_{j}\right\}$.

### 4.3. Login and Authentication Phase

In this phase, user $U_{i}$, with the assistance of GWN, exchanges a session key with $S_{j}$. The details are as follows:

1. $U_{i}$ attempts to log in by entering $ID_{i}$ and $PW_{i}$. Then, they generate random numbers $N_{i}$ and $x_{i}$. After computing $TC_{i}=B_{i}⊕h\left(ID_{i}\|PW_{i}\right)$, $U_{i}$ proceeds to calculate $T_{1}=x_{i}P$, $T_{2}=(ID_{i}\|TE_{i}\|SID_{j}\left\|N_{i}\right)⊕h\left(x_{i}pk\right)$, and $T_{3}=h(T_{1}\|ID_{i}\|ID_{GWN}\|TC_{i}\|N_{i}\|TE_{i}\left\|SID_{j}\right)$. Finally, $U_{i}$ sends the message $M_{1}=\left\{T_{1},\;T_{2},\;T_{3}\right\}$ to GWN via a public channel.
  (1) $$T_{1}=x_{i}P$$
  (2) $$T_{2}=(ID_{i}\|TE_{i}\|SID_{j}\left\|N_{i}\right)⊕h\left(x_{i}pk\right)$$
  (3) $$T_{3}=h(T_{1}\|ID_{i}\|ID_{GWN}\|TC_{i}\|N_{i}\|TE_{i}\left\|SID_{j}\right)$$
2. GWN receives $M_{1}=\left\{T_{1},\;T_{2},\;T_{3}\right\}$ from $U_{i}$ and performs the following computations to authenticate $U_{i}$: $(ID_{i}\|TE_{i}\|SID_{j}\left\|N_{i}\right)=T_{2}⊕h\left(x_{i}pk\right)$, $TC_{i}=h(ID_{i}\|ID_{GWN}\|pk\|TE_{i})$, and $T_{3}=h(T_{1}\|ID_{i}\|ID_{GWN}\|TC_{i}\|N_{i}\|TE_{i}\left\|SID_{j}\right)$. If the $T_{3}=T_{3}$ values match and $TE_{i}$ is also correct, GWN generates $N_{2},$$x_{1}$, and $x_{2}$ and then performs the following computations: $TC_{j}=h\left(K_{GS}\|SID_{j}\right)$, $T_{4}=x_{2}⊕h(TC_{j}\|N_{2}\left\|ID_{GWN}\right)$, $T_{5}=h(ID_{i}\|TE_{i}\left\|x\right)⊕h\left(N_{2}\|TC_{j}\right)$, and $T_{6}=h(T_{1}\|h(ID_{i}\|TE_{i}\left\|x)\right\|x_{2}\left\|N_{2}\right)$. Finally, GWN sends $M_{2}=\left\{T_{1},\;T_{4},\;T_{5},\;T_{6},\;N_{2}\right\}$ to $S_{j}$ through a public channel.
  (4) $$T_{4}=x_{2}⊕h(TC_{j}\|N_{2}\left\|ID_{GWN}\right)$$
  (5) $$T_{5}=h(ID_{i}\|TE_{i}\left\|x\right)⊕h\left(N_{2}\|TC_{j}\right)$$
  (6) $$T_{6}=h(T_{1}\|h(ID_{i}\|TE_{i}\left\|x)\right\|x_{2}\left\|N_{2}\right)$$
3. $S_{j}$ receives $M_{2}=\left\{T_{1},\;T_{4},\;T_{5},\;T_{6},\;N_{2}\right\}$ from GWN and performs the following computations: $x_{2}=T_{4}⊕h(TC_{j}\|N_{2}\left\|ID_{GWN}\right)$, $h(ID_{i}\|TE_{i}\left\|x\right)=T_{5}⊕h\left(N_{2}\|TC_{j}\right)$, and $T_{6}=h(T_{1}\|h(ID_{i}\|TE_{i}\left\|x)\right\|x_{2}\left\|N_{2}\right)$. $S_{j}$ verifies if $T_{6}=T_{6}$ and then generates $N_{3}$ and $x_{3}$. Afterward, $S_{j}$ performs the following computations: $T_{7}=x_{3}P$, $SK=h(h(ID_{i}\|TE_{i}\|x)\|SID_{j}\|x_{3}T_{1}\|T_{1}\left\|T_{7}\right)$, $T_{8}=h\left(SK\|N_{3}\right)$, $T_{9}=(T_{8}\|T_{7}\left\|N_{3}\right)⊕h\left(TC_{j}\|N_{2}\right)$, and $T_{10}=h(TC_{j}\|T_{7}\|N_{2}\left\|T_{8}\right)$. Finally $S_{j}$ sends $M_{3}=\left\{T_{9},\;T_{10}\right\}$ to GWN via a public channel.
  (7) $$T_{7}=x_{3}P$$
  (8) $$SK=h(h(ID_{i}\|TE_{i}\|x)\|SID_{j}\|x_{3}T_{1}\|T_{1}\left\|T_{7}\right)$$
  (9) $$T_{8}=h\left(SK\|N_{3}\right)$$
  (10) $$T_{9}=(T_{8}\|T_{7}\left\|N_{3}\right)⊕h\left(TC_{j}\|N_{2}\right)$$
  (11) $$T_{10}=h(TC_{j}\|T_{7}\|N_{2}\left\|T_{8}\right)$$
4. GWN receives $M_{3}=\left\{T_{9},\;T_{10}\right\}$ and performs the following computations: $(T_{8}\|T_{7}\left\|N_{3}\right)=T_{9}⊕h\left(TC_{j}\|N_{2}\right)$ and $T_{10}=h(TC_{j}\|T_{7}\|N_{2}\left\|T_{8}\right)$. Then, GWN verifies if $T_{10}=T_{10}$ and computes $T_{11}=(T_{8}\|N_{1}\|T_{7}\|N_{3}\left\|x\right)⊕h\left(N_{1}\|TC_{j}\right)$. Subsequently, GWN sends $M_{4}=\left\{T_{11}\right\}$ to $U_{i}$ via a public channel.
  (12) $$T_{11}=(T_{8}\|N_{1}\|T_{7}\|N_{3}\left\|x\right)⊕h\left(N_{1}\|TC_{j}\right)$$
5. $U_{i}$ receives $M_{4}=\left\{T_{11}\right\}$ from GWN and performs the following computations: $(T_{8}\|N_{1}\|T_{7}\|N_{3}\left\|x\right)=T_{11}⊕h\left(N_{1}\|TC_{j}\right)$, $SK=h(h(ID_{i}\|TE_{i}\|x)\|SID_{j}\|x_{1}T_{7}\|T_{1}\left\|T_{7}\right)$, and $T_{8}=h\left(SK\|N_{3}\right)$. Then, $U_{i}$ verifies if $T_{8}=T_{8}$.
  (13) $$SK=h(h(ID_{i}\|TE_{i}\|x)\|SID_{j}\|x_{1}T_{7}\|T_{1}\left\|T_{7}\right)$$

## 5. Limitations of Hu et al.’s Scheme

We discovered a critical vulnerability in Hu et al. [8]’s scheme. First, if an attacker gains control of a sensor node, the attacker can extract the critical values necessary for authentication. Subsequently, the attacker can generate values as if they were sensor nodes, causing confusion in the gateway node. Ultimately, an attacker can share the session key with the user, rendering it legitimate. A detailed explanation of the vulnerability found in Hu et al. [8]’s scheme is provided in the following sections.

### 5.1. Extraction of Critical Authentication Values

The attacker can extract information from the public channel and obtain data from the sensor node, allowing the attacker to capture $M_{2}=\left\{T_{1},\;T_{4},\;T_{5},\;T_{6},\;N_{2}\right\}$ and acquire the information $\left\{SID_{j},\;TC_{j}\right\}$ of sensor node $S_{j}$. Because $\left\{SID_{j},\;TC_{j}\right\}$ contains all the data $S_{j}$ may hold, the attacker gains access to both $M_{2}=\left\{T_{1},\;T_{4},\;T_{5},\;T_{6},\;N_{2}\right\}$ and $\left\{SID_{j},\;TC_{j}\right\}$.

### 5.2. Impersonation of Sensor Nodes

The attacker masquerades as the sensor node $S_{j}$, generates $N_{3}^{\prime }$ and $x_{3}^{\prime }$, and then performs the following computations: $x_{2}=T_{4}⊕h(TC_{j}\|N_{2}\left\|ID_{GWN}\right)$, $h(ID_{i}\|TE_{i}\left\|x\right)=T_{5}⊕h\left(N_{2}\|TC_{j}\right)$, $T_{6}=h(T_{1}\|h(ID_{i}\|TE_{i}\left\|x)\right\|x_{2}\left\|N_{2}\right)$, $T_{7}^{\prime }=x_{3}^{\prime }P$, $SK^{\prime }=h(h(ID_{i}\|TE_{i}\|x)\|SID_{j}\|x_{3}^{\prime }T_{1}\|T_{1}\left\|T_{7}^{\prime }\right)$, $T_{8}^{\prime }=h\left(SK^{\prime }\|N_{3}^{\prime }\right)$, $T_{9}^{\prime }=(T_{8}^{\prime }\|T_{7}^{\prime }\left\|N_{3}^{\prime }\right)⊕h\left(TC_{j}\|N_{2}\right)$, and $T_{10}^{\prime }=h(TC_{j}\|T_{7}^{\prime }\|N_{2}\left\|T_{8}^{\prime }\right)$. The attacker then pretends to be $S_{j}$ and sends $M_{3}^{\prime }=\left\{T_{9}^{\prime },\;T_{10}^{\prime }\right\}$ to GWN via a public channel.

Subsequently, GWN receives $M_{3}^{\prime }=\left\{T_{9}^{\prime },\;T_{10}^{\prime }\right\}$ from the attacker masquerading as $S_{j}$ and performs the following computations: $(T_{8}^{\prime }\|T_{7}^{\prime }\left\|N_{3}^{\prime }\right)=T_{9}^{\prime }⊕h\left(TC_{j}\|N_{2}\right)$ and $T_{10}^{\prime \prime }=h(TC_{j}\|T_{7}^{\prime }\|N_{2}\left\|T_{8}^{\prime }\right)$. It verifies if $T_{10}^{\prime }=T_{10}^{\prime \prime }$, computes $T_{11}^{\prime }=(T_{8}^{\prime }\|N_{1}\|T_{7}^{\prime }\|N_{3}^{\prime }\left\|x\right)⊕h\left(N_{1}\|TC_{j}\right)$, and then sends $M_{4}^{\prime }=\left\{T_{11}^{\prime }\right\}$ to $U_{i}$ via a public channel.

### 5.3. Illegitimate Session Key Exchange

$U_{i}$ receives $M_{4}^{\prime }=\left\{T_{11}^{\prime }\right\}$ from GWN and performs the following computations: $(T_{8}^{\prime }\|N_{1}\|T_{7}^{\prime }\|N_{3}^{\prime }\left\|x\right)=T_{11}^{\prime }⊕h\left(N_{1}\|TC_{j}\right)$ and $SK^{\prime }=h(h(ID_{i}\|TE_{i}\|x)\|SID_{j}\|x_{1}T_{7}^{\prime }\|T_{1}\left\|T_{7}^{\prime }\right)$. At this point, because the equation $x_{1}T_{7}^{\prime }=x_{1}x_{3}^{\prime }P=x_{3}^{\prime }T_{1}$ holds true, $SK^{\prime }$ is the session key shared by an attacker impersonating $S_{j}$.

## 6. Proposed Scheme

**Figure 1:**
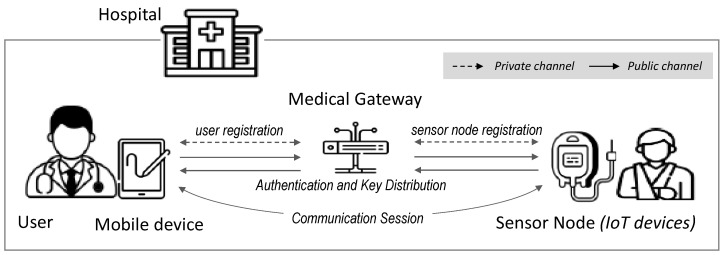
Our protocol.

The proposed scheme consists of four phases: user registration, sensor node registration, authentication, key distribution, and password update. The notation used in the protocol design is defined in Table 1, and the proposed scheme is illustrated in Figure 1.

### 6.1. User Registration Phase

**Figure 2:**
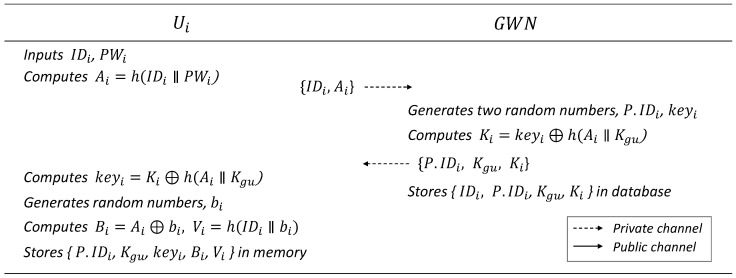
User registration phase.

In this phase, the user registers their identity and password and issues a smart card for login. The details are presented in Figure 2.

1. User $U_{i}$ enters $ID_{i}$ and $PW_{i}$ into the mobile device and computes $A_{i}=h\left(ID_{i}\|PW_{i}\right)$. Then, Ui sends the message $\left\{ID_{i},A_{i}\right\}$ to GWN through a private channel.
  (14) $$A_{i}=h\left(ID_{i}\|PW_{i}\right)$$
2. GWN generates two random numbers $P.ID_{i},key_{i}$ after receiving the message from $U_{i}$ and computes $K_{i}=key_{i}⊕h\left(A_{i}\|K_{gu}\right)$. Then, it stores $\left\{ID_{i},P.ID_{i},K_{gu},Ki\right\}$ in the database and sends the message $\left\{P.ID_{i},K_{gu},K_{i}\right\}$ to $U_{i}$ through a private channel.
  (15) $$K_{i}=key_{i}⊕h\left(A_{i}\|K_{gu}\right)$$
3. $U_{i}$ generates a random number $b_{i}$ after receiving the message from GWN, computes $key_{i}=K_{i}⊕h\left(A_{i}\|K_{gu}\right)$ and $B_{i}=A_{i}⊕b_{i}$, $V_{i}=h\left(ID_{i}\|b_{i}\right)$, and stores $\left\{P.ID_{i},K_{gu},B_{i},V_{i}\right\}$ in the memory of $U_{i}$’s mobile device.
  (16) $$key_{i}=K_{i}⊕h\left(A_{i}\|K_{gu}\right)$$
  (17) $$B_{i}=A_{i}⊕b_{i}$$
  (18) $$V_{i}=h\left(ID_{i}\|b_{i}\right)$$

### 6.2. Sensor Node Registration Phase

**Figure 3:**
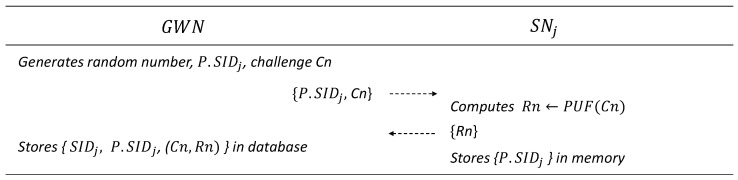
Sensor registration phase.

A sensor node must be registered using GWN. The details are presented in Figure 3.

1. GWN generates a random number $P.SID_{j}$ and a challenge $C_{n}$ and sends them to the sensor node $SN_{j}$ through a private channel.
2. $SN_{j}$ outputs the response $R_{n}<-PUF\left(Cn\right)$ and sends it to GWN through a private channel.
3. $SN_{j}$ stores $P.SID_{j}$ in the memory.

### 6.3. Authentication and Key Distribution Phase

**Figure 4:**
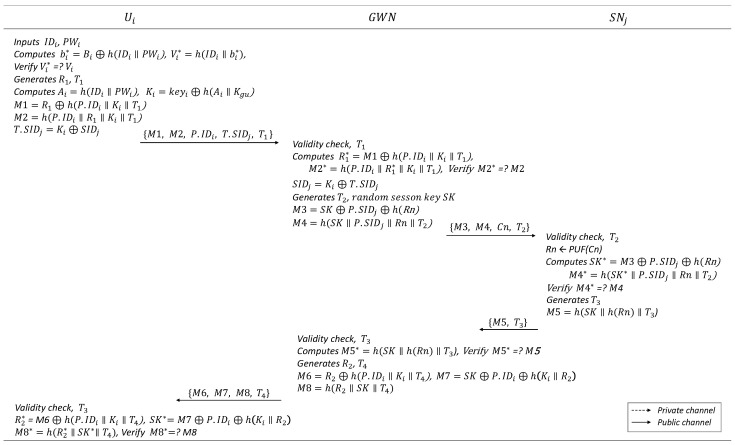
Login and authentication phase.

In the authentication and key distribution phases, when user $U_{i}$ logs in, GWN verifies it and facilitates the exchange of the session key between the user and sensor node $SN_{j}$. The details are presented in Figure 4.

1. $U_{i}$ inputs $ID_{i}$ and $PW_{i}$ to the mobile device, which computes $b_{i}^{*}=B_{i}⊕h\left(ID_{i}\|PW_{i}\right)$ and $V_{i}^{*}=h\left(ID_{i}\|b_{i}^{*}\right)$. Then, the device verifies whether $V_{i}^{*}=V_{i}$. If it does not match, the device stops the protocol; otherwise, it proceeds to the next steps. In this process, a method of limiting the maximum number of login attempts using a counter can be used to consider the case where an attacker attempts multiple logins. For example, if the counter is set to 10, if an attacker attempts to login more than 10 times, the login function on the mobile device can be locked for a certain period of time.
2. $U_{i}$ generates a random number $R_{1}$ and timestamp $T_{1}$ and computes $A_{i}=h\left(ID_{i}\|PW_{i}\right)$, $K_{i}=key_{i}⊕h\left(A_{i}\|K_{gu}\right)$, $M1=R_{1}⊕h(P.ID_{i}\|K_{i}\left\|T_{1}\right)$, $M2=h(P.ID_{i}\|R_{1}\|K_{i}\|T_{1})$, and $T.SID_{j}=K_{i}⊕SID_{j}$. Subsequently, $U_{i}$ sends the message $\left\{M1,\;M2,\;P.ID_{i},\;T.SID_{j},\;T_{1}\right\}$ to GWN.
  (19) $$M1=R_{1}⊕h(P.ID_{i}\|K_{i}\left\|T_{1}\right)$$
  (20) $$M2=h(P.ID_{i}\|R_{1}\|K_{i}\|T_{1})$$
  (21) $$T.SID_{j}=K_{i}⊕SID_{j}$$
3. After receiving the message from $U_{i}$, GWN first checks the validity of the timestamp $T_{1}$. If it is not valid, GWN stops the protocol; otherwise, GWN checks $P.ID_{i}$ in the message and computes $R_{1}^{*}=M1⊕h(P.ID_{i}\|K_{i}\left\|T_{1}\right)$ and $M2^{*}=h(P.ID_{i}\|R_{1}^{*}\|K_{i}\left\|T_{1}\right)$ using the information stored in the database for $U_{i}$, then verifies whether $M_{2}^{*}=M_{2}$.
4. GWN computes $SID_{j}=K_{i}⊕T.SID_{j}$ and checks the $SID_{j}$ of $SN_{j}$.
5. Then, GWN generates the random session key SK and timestamp $T_{2}$ and computes $M_{3}=SK⊕P.SID_{j}⊕h\left(Rn\right)$ and $M4=h(SK\|P.SID_{j}\|Rn\|T_{2})$. Then, GWN sends the message $\left\{M3,M4,Cn,T_{2}\right\}$ to $SN_{j}$.
  (22) $$SID_{j}=K_{i}⊕T.SID_{j}$$
  (23) $$M_{3}=SK⊕P.SID_{j}⊕h\left(Rn\right)$$
  (24) $$M4=h(SK\|P.SID_{j}\|Rn\|T_{2})$$
6. $SN_{j}$ checks the validity of the timestamp $T_{2}$. Then, $SN_{j}$ outputs the response Rn<−PUF(Cn), using the challenge Cn received from GWN. $SN_{j}$ computes $SK^{*}=M3⊕P.SID_{j}⊕h\left(Rn\right)$ and $M4*=h(SK^{*}\|P.SID_{j}\|Rn\|T_{2})$, and then verifies whether $M4^{*}=M4$.
  (25) $$SK^{*}=M3⊕P.SID_{j}⊕h\left(Rn\right)$$
7. $SN_{j}$ generates timestamp T3 and computes $M5=h(SK\|h\left(Rn\right)\|T_{3})$. Then, $SN_{j}$ sends the message $\left\{M5,T_{3}\right\}$ to GWN.
  (26) $$M5=h(SK^{*}\|h\left(Rn\right)\|T_{3})$$
8. GWN checks the validity of timestamp $T_{3}$. If it is not valid, GWN stops the protocol; otherwise, GWN computes $M5^{*}=h(SK\|h\left(Rn\right)\|T_{3})$ and then verifies whether $M5^{*}=M5$.
9. GWN generates a random number $R_{2}$ and timestamp $T_{3}$, and computes $M6=R_{2}⊕h(P.ID_{i}\|K_{i}\left\|T_{4}\right)$, $M7=SK⊕P.ID_{i}⊕h\left(K_{i}\|R_{2}\right)$, and $M8=h(R_{2}\|SK\|T_{4})$. Then, GWN sends the message $\left\{M6,M7,M8,T_{4}\right\}$ to $U_{i}$.
  (27) $$M6=R_{2}⊕h(P.ID_{i}\|K_{i}\left\|T_{4}\right)$$
  (28) $$M7=SK⊕P.ID_{i}⊕h\left(K_{i}\|R_{2}\right)$$
  (29) $$M8=h(R_{2}\|SK\|T_{4})$$
10. Ui checks the validity of the timestamp $T_{4}$. If it is not valid, the GWN stops the protocol; otherwise, Ui computes $R_{2}^{*}=M6⊕h(P.ID_{i}\|K_{i}\left\|T_{4}\right)$, $SK^{*}=M7⊕P.ID_{i}⊕h\left(K_{i}\|R_{2}\right)$, and $M8^{*}=h(R_{2}^{*}\|SK^{*}\left\|T_{4}\right)$. Then, it verifies whether $M8^{*}=M8$.
  (30) $$SK^{*}=M7⊕P.ID_{i}⊕h\left(K_{i}\|R_{2}\right)$$

Once all the processes are completed, the authentication and key distribution between $U_{i}$ and $SN_{j}$ through GWN are completed. Before terminating the session, $U_{i}$ and the GWN update the new user pseudonym using $R_{2}$ as follows: $P.ID_{i}^{n}=P.ID_{i}⊕R_{2}$ and $P.ID_{i}=P.ID_{i}^{n}$. In addition, GWN and $SN_{j}$ update the new sensor node pseudonym using SK as follows: $P.SID_{j}^{n}=P.SID_{j}⊕h\left(SK\right)$ and $P.SID_{j}=P.SID_{j}^{n}$.

### 6.4. Password Update Phase

**Figure 5:**
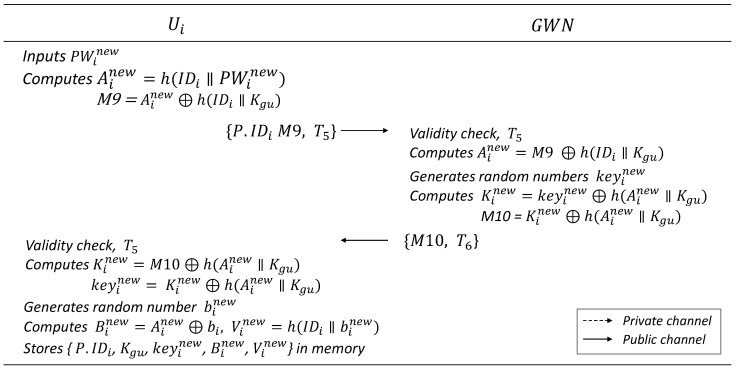
Password change phase.

Users must be able to change their passwords when required. The proposed scheme allows the users to change their password with the help of GWN, as shown in Figure 5.

1. $U_{i}$ inputs a new password, $PW_{i}^{n}$, and computes $A_{i}^{n}ew=h\left(ID_{i}\|PW_{i}^{n}\right)$ and $M9=A_{i}^{n}⊕h\left(ID_{i}\|K_{gu}\right)$. $U_{i}$ sends the message {P.ID_i,M9} to GWN.
2. GWN computes $A_{i}^{n}=M9⊕h\left(ID_{i}\|K_{gu}\right)$ and generates a random number $key_{i}^{n}$. Subsequently, GWN computes $K_{i}^{n}=key_{i}^{n}⊕h\left(A_{i}^{n}\|K_{gu}\right)$ and $M10=K_{i}^{n}⊕h\left(A_{i}^{n}\|K_{gu}\right)$, and sends the message {M10} to $U_{i}$.
3. $U_{i}$ computes $K_{i}^{n}=M10⊕h\left(A_{i}^{n}\|K_{gu}\right)$ and $key_{i}^{n}=K_{i}^{n}⊕h\left(A_{i}^{n}\|K_{gu}\right)$ and generates a random number $b_{i}^{n}ew$. Subsequently, $U_{i}$ computes $B_{i}^{n}=A_{i}^{n}⊕b_{i},V_{i}^{n}=h\left(ID_{i}\|b_{i}^{n}\right)$.
4. $U_{i}$ stores $\left\{P.ID_{i},K_{gu},key_{i}^{n},B_{i}^{n},V_{i}^{n}\right\}$ in the memory.

## 7. Security Analysis of the Proposed Scheme

In this section, we describe the formal security analysis conducted using ProVerif as well as an informal security analysis addressing seven key security properties. The details are as follows:

### 7.1. Formal Security Analysis

ProVerif is a tool for automatically analyzing the security of cryptographic protocols. ProVerif (1) checks whether specific values in the protocol are not exposed to attackers, (2) verifies that mutual trust relationships between communication participants are properly established, and (3) analyzes traceability and anonymity, providing analysis for protocols with unlimited sessions and an unlimited message space [20].

We verified the proposed scheme using ProVerif, a formal analysis tool that ensures the proper functioning of programs. As in several recent studies [21,22,23], we used ProVerif to demonstrate that our scheme provides a viable authentication method and prevents the extraction of sensitive information.

We verify the results in Table 2 using the queries listed in Table 3. The results are as follows:

- Query inj-event(EVENT) ==> inj-event(EVENT) is true.
- Query not attacker(K) is true.

The statement “Query inj-event(EVENT) ==> inj-event(EVENT) is true” confirms that the event has been successfully verified, signifying that the event occurred as intended. This implies that, under the specified conditions, the authentication mechanism operates as expected. Similarly, the statement “Query not attacker(K) is true” indicates that the attacker was unable to obtain the keys within the array, as the query result confirms the security of the keys.

For the registration phase, we employed two private channels, and for the login and authentication phases, we used two public channels. Each channel is assumed to connect user Ui and the GWN, as well as the GWN and sensor node SNj. Specifically, privateChannel1 serves as the private channel linking Ui and the GWN during the registration phase for Ui. Similarly, privateChannel2 connects SNj and the GWN during the registration phase for SNj. For the login and authentication phase, publicChannel1 links Ui to the GWN, while publicChannel2 connects SNj to the GWN.

To verify the security of the session key, we define it as a private variable. We also predefine information, such as IDi, PWi, Kgu, GWNd, SIDj, and SID. The identifiers IDi, GWNd, and SID were separately defined to distinguish individuals accessing the system during the login and authentication phases. We define a concat function responsible for the concatenation, hash function, PUF function, and XOR operation, all of which are integral to the system. Additionally, we define an event that distinguishes between the login and authentication processes. These definitions are summarized in Table 4.

The behavioral information for each Ui, GWN, and SNj during the registration, login, and authentication phases is presented in Table 5, Table 6 and Table 7, respectively. It has been explicitly stated that the registration phase occurs through private channels, whereas the login and authentication phases occur via public channels.

### 7.2. Informal Security Analysis

The proposed scheme satisfied seven critical security requirements. The security properties we verified were impersonation attacks, anonymity and untraceability, replay attacks, forward secrecy, mutual authentication, man-in-the-middle attacks, and PUF modeling attacks. We summarize the latest studies and indicate whether they satisfy the corresponding security properties in Table 8. The detailed explanations of each security property are as follows.

#### 7.2.1. Impersonation Attack

In our proposed scheme, even if the attackers physically obtain the user’s mobile device or sensor node and capture the stored information, they cannot impersonate a legitimate user $U_{i}$ or a sensor node $SN_{j}$. The information stored in the user’s mobile device is $\left\{P.ID_{i},\;K_{gu},\;key_{i},\;B_{i},\;V_{i}\right\}$. Even if the attackers know this information, they cannot log into the mobile device unless they input a legitimate $PW_{i}$. Even if the attackers log into the mobile device, they cannot proceed with the user authentication procedure because they cannot generate the $A_{i}$ shared with GWN during the user registration phase without a legitimate $PW_{i}$. Even if the attackers obtain $SN_{j}$, the only information stored in $SN_{j}$ is $P.SID_{j}$, and even if they use the trace transmitted through the public channel, they cannot generate the response Rn used as authentication information because of the PUF characteristics. Thus, they cannot proceed with the sensor node authentication procedure.

#### 7.2.2. Anonymity and Untraceability

In the authentication and key distribution phase, $ID_{i}$ and $SID_{j}$ are not transmitted over the public channel, so even if an attacker obtains the message, he cannot figure out $ID_{i}$ and $SID_{j}$. In addition, the pseudonyms of the user and sensor, $P.ID_{i}$ and $P.SID_{j}$, are randomly generated by the GWN during the user and sensor registration phase. Therefore, even if an attacker obtains the user $P.ID_{i}$ and the sensor $P.SID_{j}$, he cannot find $ID_{i}$ and $SID_{j}$. Thus, the proposed scheme provides anonymity for the user and the sensor (actually, the patient wearing the sensor). Furthermore, at the end of every session, $U_{i}$ and GWN and GWN and $SN_{j}$ update $P.ID_{i}$ and $P.SID_{j}$ to $P.ID_{i}^{n}$ and $P.SID_{j}^{n}$, respectively. In the process, $P.ID_{i}^{n}$ and $P.SID_{j}^{n}$ are generated using GWN-generated random numbers $R_{2}$ and SK, so even if an attacker captures the mobile device and sensor and obtains the $P.ID_{i}$ and $P.SID_{j}$ stored on each, he cannot trace the user and sensor to the pseudonym used per session.

#### 7.2.3. Replay Attack

All messages transmitted during the authentication and key-distribution phases include a timestamp, and entities that receive the transmitted message proceed with subsequent processes only if the timestamp is valid. Therefore, the proposed protocol can withstand replay attacks.

#### 7.2.4. Forward Secrecy

In our proposed scheme, session keys are generated randomly for each session. Therefore, even if an attacker knows or has the previous session key, the attacker cannot infer other session keys. Therefore, the proposed scheme satisfies forward secrecy.

#### 7.2.5. Mutual Authentication

During the authentication and key distribution phases, $U_{i}$, GWN, and $SN_{j}$ are mutually authenticated. GWN and $U_{i}$ authenticate each other by verifying the messages $M2^{*}=h(P.ID_{i}\|R_{1}^{*}\|K_{i}\left\|T_{1}\right)$ and $M8^{*}=h(R_{2}\|SK^{*}\left\|T_{4}\right)$, respectively. Similarly, GWN and $SN_{j}$ authenticate each other by verifying the messages $M5^{*}=h(SK\|h\left(R_{n}\right)\left\|T_{4}\right)$ and $M4^{*}=h(SK^{*}\|P.SID_{j}\|R_{n}\left\|T_{2}\right)$. If any of the verifications are invalid, the session is aborted. Therefore, the proposed scheme provides mutual authentication.

#### 7.2.6. Man-in-the-Middle Attack

Assume that an attacker can intercept all messages sent to a public channel. Here, we take the message $M_{1},M_{2},P.ID_{i},T.SID_{j},T_{1}$ sent by $U_{i}$ to GWN during the authentication phase as an example. An attacker attempts to alter the authentication value $M_{2}=h(P.ID_{i}\|R_{1}\|K_{i}\left\|T_{1}\right)$. However, the attacker does not know $K_{i}$ and $R_{1}$, and therefore cannot compute $M_{2}$. Thus, the request message sent by the attacker cannot be authenticated using GWN. Similarly, M4, M5, and M8, which are used as authentication values, contain information that is unknown to the attacker. As a result, tampering with authentication values is impossible. Therefore, the proposed scheme is resistant to man-in-the-middle attacks.

#### 7.2.7. PUF Modeling Attack

In the proposed scheme, during the sensor node registration phase, a challenge–response pair of the PUF [24] is transmitted over a private channel. Furthermore, during the authentication and key distribution phases, GWN sends only the challenge to $SN_{j}$. Therefore, PUF modeling attacks that collect a large number of challenge–response pairs and predict legitimate challenge–response pairs based on machine learning are not possible using the proposed scheme.

#### 7.2.8. Denial of Service Attack

During the authentication and key distribution phases, an attacker can attempt to launch denial-of-service attacks on mobile devices and sensor nodes. However, the proposed scheme can prevent denial-of-service attacks by limiting the number of attacks on the device by using counters to limit the maximum number of login attempts and timestamps to limit the possible time of attacks on sensor nodes.

## 8. Performance Analysis of the Proposed Scheme

We compared our scheme with four recent schemes [8,9,10,11] in terms of computational efficiency. We analyzed the computational load according to the experimental environment of Kim et al. [25] and He et al. [26]. The CPU used was an Intel(R) Core(TM) i7-8565U @1.80 GHz, 1.99 GHz, with 16.0 GB of RAM, and the experiments were conducted on a desktop running Windows 10 Home. The Java Development Kit (JDK) 17 was used, and elliptic curve multiplication operations were measured using the secp521r1 ECC. Extracting biometric information using a fuzzy extractor is similar to multiplying the operation time [26].

The computational times we established are as follows. We set the time required to compute a hash function to 0.007 ms, and the time for extracting biometric information from the fuzzy extractor and performing multiplication operations on the elliptic curve to 34.32 ms. Additionally, the time required to compute a symmetric key was set to 0.23 ms. The computational times for each operation are listed in Table 9.

We measured the computational overhead in the login and authentication phases of each scheme [8,9,10,11] and the proposed scheme. The results are presented in Table 10.

In Wu et al. [9]’s scheme, the user performs eight hash operations, the gateway node performs nine hash operations, and the sensor node performs five hash operations, resulting in twenty-two hash operations. This amounts to a computational cost of $22T_{h}$ = 0.154 ms.

In Sahoo et al. [10]’s scheme, the user performs six hash operations, one fuzzy extractor operation for extracting biometric data, one symmetric encryption, one symmetric decryption, and one elliptic curve multiplication. The gateway node performs five hash operations, one symmetric encryption, one symmetric decryption, and two elliptic curve multiplications. The sensor node performs six hash operations, one symmetric encryption, one symmetric decryption, and one elliptic curve multiplication. Therefore, the computational cost of Sahoo et al.’s scheme is $17T_{h}+1T_{B}+3T_{D}+3T_{E}+4T_{M}$ = 173.099 ms, which includes 17 hash operations, one fuzzy extractor, three symmetric encryptions, three symmetric decryptions, and four elliptic-curve multiplications.

In Hu et al. [8]’s scheme, the user performs six hash operations and one elliptic curve multiplication, the gateway node performs eleven hash operations, and the sensor node performs one hash operation and one elliptic curve multiplication. This results in 18 hash operations and two elliptic curve multiplications, amounting to $18T_{h}+2T_{M}$ = 68.766 ms of computational cost.

In Wenfeng Huang [11]’s scheme, the user performs 17 hash operations, four elliptic curve multiplications, and one fuzzy extractor operation; the gateway node performs 16 hash operations and two elliptic curve multiplications; and the sensor node performs 8 hash operations and two elliptic curve multiplications. This results in 41 hash operations, eight elliptic curve multiplications, and one fuzzy extractor operation, with a computational cost of $41T_{h}+8T_{M}+1T_{B}$ = 309.167 ms.

Finally, in the proposed scheme, the user performs nine hash operations, the gateway node performs eight hash operations, and the sensor node performs three hash operations, resulting in twenty hash operations at a computational cost of $20T_{h}$ = 0.14 ms.

## 9. Discussion

We compared the security analysis of state-of-the-art schemes [8,9,10,11] with that of our proposed scheme and evaluated the computational cost in terms of performance. The proposed scheme not only satisfies seven critical security properties but also demonstrates robustness against PUF modeling attacks, making it more secure than other studies in this regard.

In terms of computational cost, the time required for authentication in our scheme is 0.14 ms under the environment used by Kim et al. [25]. Compared with other schemes, using the performance improvement calculation (t2−t1)/t1, where t1 is the proposed scheme and t2 represents other schemes, the following improvements were obtained: 10% compared to Wu et al. [9], 1235.42% compared to Sahoo et al. [10], 490.18% compared to Hu et al. [8], and 2207.33% compared to Wenfeng Huang [11]. This demonstrates that our scheme not only achieves an average improvement in performance of 985.73% but also performs 10% better than Wu et al. [9], which is the most lightweight among the compared schemes.

Our performance evaluation is based on the computation metrics measured by Kim et al. [25] and He et al. [26]. Therefore, differences may arise if measurements are conducted using CPUs, GPUs with different performance characteristics, or quantum computers in the future. While the computation of hash functions is very lightweight and may not show significant differences with new devices, public key operations such as elliptic curve computations could be performed more efficiently with improved hardware.

## 10. Conclusions

In this study, we analyzed Hu et al.’s scheme and found that an attacker can impersonate legitimate sensor nodes and generate illegitimate session keys using information stored in the sensor nodes and information transmitted over public channels. To overcome these vulnerabilities, our proposed scheme utilizes PUFs to make it impossible to impersonate the sensor nodes. By using PUFs, we minimize the computation of resource-constrained sensor nodes and ensure anonymity and untraceability by using pseudonyms that change from session to session instead of user and sensor node identities. In addition, an informal security analysis shows that the proposed scheme ensures forward secrecy and mutual authentication and is resistant to man-in-the-middle and PUF modeling attacks.

However, the proposed scheme can be utilized in a single-gateway environment, assuming the internal environment of a single hospital. For future expansion to a wider range of healthcare services, a multigateway environment must be considered.

## Figures and Tables

**Table 1:** Notation.

Notation	Description
$U_{i},$ $SN_{j}$	*i*-th user, *j*-th sensor node
GWN	Medical gateway node
$ID_{i},$ $P.ID_{i}$	Identity, pseudonym of *i*-th user
$SID_{j},$ $P.SID_{j}$	Identity, pseudonym of *j*-th sensor node
(Cn, Rn)	Challenge–response pair of PUF
SK	Session key
$R_{1},$ $R_{2},$ …	Random number
$T_{1},$ $T_{2},$ …	Time stamp

**Table 2:** ProVerif query results.

Verification summary:
Query inj-event(endUi(idu)) ==> inj-event(startUi(idu)) is true.
Query inj-event(endGWN(idg)) ==> inj-event(startGWN(idg)) is true.
Query inj-event(endSNj(ids)) ==> inj-event(startSNj(ids)) is true.
Query not attacker(SK[]) is true.

**Table 3:** ProVerif code for queries.

(*—-queries—-*)
query idu:bitstring; inj-event(endUi(idu)) ==> inj-event(startUi(idu)).
query idg:bitstring; inj-event(endGWN(idg)) ==> inj-event(startGWN(idg)).
query ids:bitstring; inj-event(endSNj(ids)) ==> inj-event(startSNj(ids)).
query attacker(SK).

(*—-process—-*)
process
((!Ui)|(!GWN)|(!SNj))

**Table 4:** ProVerif code for defining values and functions.

(*—-channels—-*)
free privateChannel1:channel [private].
free privateChannel2:channel [private].
free publicChannel1:channel.
free publicChannel2:channel.

(*—-constants—-*)
free IDi:bitstring [private].
free PWi:bitstring [private].
free Kgu:bitstring [private].
free GWNd:bitstring [private].
free SIDj:bitstring.
free SID:bitstring [private].

(*—-shared key—-*)
free SK:bitstring [private].

(*—-functions—-*)
fun concat(bitstring, bitstring):bitstring.
fun h(bitstring):bitstring.
fun PUF(bitstring):bitstring.
fun xor(bitstring, bitstring):bitstring.
equation forall p:bitstring, q:bitstring; xor(xor(p, q), q)=p.

(*—-events—-*)
event startUi(bitstring).
event endUi(bitstring).
event startGWN(bitstring).
event endGWN(bitstring).
event startSNj(bitstring).
event endSNj(bitstring).

**Table 5:** ProVerif code for the Ui.

(*—-Ui process—-*)
let Ui =
let Ai = h(concat(IDi, PWi)) in
out(privateChannel1,(IDi, Ai));
in(privateChannel1, (XPIDi:bitstring, XKgu:bitstring, XKi:bitstring));
let xkeyi = xor(XKi, h(concat(Ai, XKgu))) in
new bi:bitstring;
let Bi = xor(Ai, bi) in
let Vi = h(concat(IDi, bi)) in
event startUi(IDi);
new R1:bitstring;
new T1:bitstring;
let M1 = xor(R1, h(concat(concat(XPIDi, R1), T1))) in
let M2 = h(concat(concat(XPIDi, R1), concat(XKi, T1))) in
let TSIDj = xor(SIDj, XKi) in
out(publicChannel1,(M1, M2, XPIDi, TSIDj, T1));
in(publicChannel1,(XM6:bitstring, XM7:bitstring, XM8:bitstring, XT4:bitstring));
let GR2 = xor(XM6, h(concat(concat(XPIDi, XKi), XT4))) in
let GSK = xor(XM7, xor(XPIDi, h(concat(XKi, GR2)))) in
let GM8 = h(concat(concat(GR2, GSK), XT4)) in
if (GM8=XM8) then event endUi(IDi).

**Table 6:** ProVerif code for the GWN.

(*—-GWN process—-*)
let GWN =
in(privateChannel1,(XIDi:bitstring, XAi:bitstring));
new keyi:bitstring;
new PIDi:bitstring;
let Ki = xor(keyi, h(concat(XAi, Kgu))) in
out(privateChannel1, (PIDi, Kgu, Ki));
new PSIDj:bitstring;
new Cn:bitstring;
out(privateChannel2, (PSIDj, Cn));
in(privateChannel2, (XRn:bitstring));
event startGWN(GWNd);
in(publicChannel1,(XM1:bitstring, XM2:bitstring, XPIDi:bitstring, XTSIDj:bitstring, XT1:bitstring));
let GR1 = xor(XM1, h(concat(concat(XPIDi, Ki), XT1))) in
let GM2 = h(concat(concat(XPIDi, GR1), concat(Ki, XT1))) in
if (XM2=GM2) then
let SIDj = xor(XTSIDj, Ki) in
new T2:bitstring;
new SK:bitstring;
let M3 = xor(SK, xor(PSIDj, h(XRn))) in
let M4 = h(concat(concat(SK, PSIDj), concat(XRn, T2))) in
out(publicChannel2, (M3, M4, Cn, T2));
in(publicChannel2, (XM5:bitstring, XT3:bitstring));
let GM5 = h(concat(concat(SK, h(XRn)), XT3)) in
if (XM5=GM5) then
new R2:bitstring;
new T4:bitstring;
let M6 = xor(R2, h(concat(concat(PIDi, Ki), T4))) in
let M7 = xor(SK, xor(PIDi, h(concat(Ki, R2)))) in
let M8 = h(concat(concat(R2, SK), T4)) in
out(publicChannel1, (M6, M7, M8, T4));
event endGWN(GWNd).

**Table 7:** ProVerif code for the SNj.

(*—-SNj process—-*)
let SNj =
in(privateChannel2, (XPSIDj:bitstring, XCn:bitstring));
let Rn = PUF(XCn) in
out(privateChannel2, (Rn));
event startSNj(SID);
in(publicChannel2, (XM3:bitstring, XM4:bitstring, XCn:bitstring, XT2:bitstring));
let GSK = xor(XM3, xor(XPSIDj, h(Rn))) in
let GM4 = h(concat(concat(GSK, XPSIDj), concat(Rn, XT2))) in
if (XM4=GM4) then
new T3:bitstring;
let M5 = h(concat(concat(GSK, h(Rn)), T3)) in
out(publicChannel2, (M5, T3));
event endSNj(SID).

**Table 8:** Comparison of security features.

Security Features	Wu et al. [9]	Sahoo et al. [10]	Hu et al. [8]	Wenfeng Huang [11]	Ours
Resist impersonation attack	O	O	X	O	O
Provide anonymity and untraceability	O	O	O	O	O
Resist replay attack	O	O	O	O	O
Provide forward secrecy	O	O	O	O	O
Provide mutual authentication	O	O	O	O	O
Resist man-in-the-middle attack	O	O	X	O	O
PUF modeling attack	X	X	X	X	O

**Table 9:** Computation times for each operation (ms).

Symbol	Meaning	Time (ms)
$T_{h}$	Computation time for hash functions	0.007
$T_{B}$	Extraction time of biometric information in fuzzy extractors	34.32
$T_{M}$	Multiplication operation time in ECC	34.32
$T_{E}$, $T_{D}$	Computation time for symmetric key encryption and decryption	0.23

**Table 10:** Comparisons of computational costs (ms).

Entity	Wu et al. [9]	Sahoo et al. [10]	Hu et al. [8]	Wenfeng Huang [11]	Ours
User	$8T_{h}$ = 0.056	$6T_{h}+1T_{B}+1T_{E}+1T_{D}+1T_{M}$ = 69.142	$6T_{h}+1T_{M}$ = 34.362	$17T_{h}+4T_{M}+1T_{B}$ = 171.719	$9T_{h}$ = 0.063
Gateway node	$9T_{h}$ = 0.063	$5T_{h}+1T_{D}+1T_{E}+2T_{M}$ = 69.135	$11T_{h}$ = 0.077	$16T_{h}+2T_{M}$ = 68.696	$8T_{h}$ = 0.056
Sensor node	$5T_{h}$ = 0.035	$6T_{h}+1T_{D}+1T_{E}+1T_{M}$ = 34.822	$1T_{h}+1T_{M}$ = 34.327	$8T_{h}+2T_{M}$ = 68.696	$3T_{h}$ = 0.021
Total	$22T_{h}$ = 0.154	$17T_{h}+1T_{B}+3T_{D}+3T_{E}+4T_{M}$ = 173.099	$18T_{h}+2T_{M}$ = 68.766	$41T_{h}+8T_{M}+1T_{B}$ = 309.167	$20T_{h}$ = 0.14

## Data Availability

Data are contained within the article.

## References

[1] Ekambaram D., Ponnusamy V. (2023). AI-assisted Physical Therapy for Post-injury Rehabilitation: Current State of the Art. IEIE Trans. Smart Process. Comput..

[2] Gawali V., Pande M., Sayyad M., Bhadade R. (2024). An Automated Drainage Vision-based Monitoring System-ADVMS: Key Component in Developing Smart Cities in India. IEIE Trans. Smart Process. Comput..

[3] Shojaei P., Vlahu-Gjorgievska E., Chow Y.W. (2024). Security and privacy of technologies in health information systems: A systematic literature review. Computers.

[4] Keshta I., Odeh A. (2021). Security and privacy of electronic health records: Concerns and challenges. Egypt. Inform. J..

[5] Mbonihankuye S., Nkunzimana A., Ndagijimana A. (2019). Healthcare data security technology: HIPAA compliance. Wirel. Commun. Mob. Comput..

[6] Peng S., Zhao L., Al-Dubai A.Y., Zomaya A.Y., Hu J., Min G., Wang Q. (2021). Secure lightweight stream data outsourcing for internet of things. IEEE Internet Things J..

[7] Kumar P., Chouhan L. (2021). A privacy and session key based authentication scheme for medical IoT networks. Comput. Commun..

[8] Hu B., Tang W., Xie Q. (2022). A two-factor security authentication scheme for wireless sensor networks in IoT environments. Neurocomputing.

[9] Wu T.Y., Wang L., Chen C.M. (2023). Enhancing the security: A lightweight authentication and key agreement protocol for smart medical services in the ioht. Mathematics.

[10] Sahoo S.S., Mohanty S., Sahoo K.S., Daneshmand M., Gandomi A.H. (2023). A three-factor-based authentication scheme of 5G wireless sensor networks for IoT system. IEEE Internet Things J..

[11] Huang W. (2024). ECC-based three-factor authentication and key agreement scheme for wireless sensor networks. Sci. Rep..

[12] Ostad-Sharif A., Arshad H., Nikooghadam M., Abbasinezhad-Mood D. (2019). Three party secure data transmission in IoT networks through design of a lightweight authenticated key agreement scheme. Future Gener. Comput. Syst..

[13] Chen C.T., Lee C.C., Lin I.C. (2020). Efficient and secure three-party mutual authentication key agreement protocol for WSNs in IoT environments. PLoS ONE.

[14] Zhang L., Zhang Y., Tang S., Luo H. (2018). Privacy protection for e-health systems by means of dynamic authentication and three-factor key agreement. IEEE Trans. Ind. Electron..

[15] Aghili S.F., Mala H., Shojafar M., Peris-Lopez P. (2019). LACO: Lightweight three-factor authentication, access control and ownership transfer scheme for e-health systems in IoT. Future Gener. Comput. Syst..

[16] Amintoosi H., Nikooghadam M., Shojafar M., Kumari S., Alazab M. (2022). Slight: A lightweight authentication scheme for smart healthcare services. Comput. Electr. Eng..

[17] Guo H., Gao Y., Xu T., Zhang X., Ye J. (2019). A secure and efficient three-factor multi-gateway authentication protocol for wireless sensor networks. Ad Hoc Netw..

[18] Xue L., Huang Q., Zhang S., Huang H., Wang W. (2021). A lightweight three-factor authentication and key agreement scheme for multigateway WSNs in IoT. Secur. Commun. Netw..

[19] Schläpfer T., Rüst A. Security on IoT devices with secure elements. Proceedings of the Embedded World Conference.

[20] Blanchet B., Smyth B., Cheval V., Sylvestre M. (2018). ProVerif 2.00: Automatic Cryptographic Protocol Verifier. User Man. Tutor..

[21] Lee H., Ryu J. (2024). Physical-Unclonable-Function-based Secure and Anonymous User Authentication for Smart Homes. IEEE Access.

[22] Kang T., Woo N., Ryu J. (2024). Enhanced Lightweight Medical Sensor Networks Authentication Scheme Based on Blockchain. IEEE Access.

[23] Kook S., Kim K., Ryu J., Lee Y., Won D. (2024). Lightweight Hash-Based Authentication Protocol for Smart Grids. Sensors.

[24] Rührmair U., Sölter J., Sehnke F., Xu X., Mahmoud A., Stoyanova V., Dror G., Devadas S. (2013). PUF modeling attacks on simulated and silicon data. IEEE Trans. Inf. Forensics Secur..

[25] Kim K., Ryu J., Lee H., Lee Y., Won D. (2023). Distributed and Federated Authentication Schemes Based on Updatable Smart Contracts. Electronics.

[26] He D., Kumar N., Lee J.H., Sherratt R.S. (2014). Enhanced three-factor security protocol for consumer USB mass storage devices. IEEE Trans. Consum. Electron..

